# *In-situ* Liquid Phase Epitaxy: Another Strategy to Synthesize Heterostructured Core-shell Composites

**DOI:** 10.1038/srep25260

**Published:** 2016-04-28

**Authors:** Zhongsheng Wen, Guanqin Wang

**Affiliations:** 1 Department of Materials, Dalian Maritime University, 116026 Dalian, China

## Abstract

Core-shell Nb_2_O_5_/TiO_2_ composite with hierarchical heterostructure is successfully synthesized *In-situ* by a facile template-free and acid-free solvothermal method based on the mechanism of liquid phase epitaxy. The chemical circumstance change induced by the alcoholysis of NbCl_5_ is utilized tactically to trigger core-shell assembling *In-situ*. The tentative mechanism for the self-assembling of core-shell structure and hierarchical structure is explored. The microstructure and morphology changes during synthesis process are investigated systematically by using X-ray diffraction, scanning electron microscopy, X-ray photoelectron spectroscopy and transmission electron microscopy. The dramatic alcoholysis of NbCl_5_ has been demonstrated to be the fundamental factor for the formation of the spherical core, which changes the acid circumstance of the solution and induces the co-precipitation of TiO_2_. The homogeneous co-existence of Nb_2_O_5_/TiO_2_ in the core and the co-existence of Nb/Ti ions in the reaction solution facilitate the *In-situ* nucleation and epitaxial growth of the crystalline shell with the same composition as the core. *In-situ* liquid phase epitaxy can offer a different strategy for the core-shell assembling for oxide materials.

The development of nanostructured metal oxide with controlled morphology has triggered intensive interests because of the intimate intercorrelation between the morphology/microstructure and the performance. Microstructure controlling via stereo structure building and preferential orientation growing has been demonstrated a very efficient way to tune the final performance for versatile catalysts^1^,^2^,^3^,^4^,^5^,^6^,^7^.

Core-shell and hollow microstructures have received aggressive attention in many areas of science and technology for their special properties different from bulk and homogeneous nanomaterials. Generally, core-shell structure can be synthesized by cost-effective method based on chemical precipitation or the mechanism of Ostwald Ripening^8^,^9^,^10^, Kirkendall effect^11^,^12^,^13^, or orientation attachment^14^,^15^. In such synthesizing process, exotic template (or template precursor) is generally necessary to be introduced as core template (or core precursors) to configure core-shell architecture. The surface of the core template with different functional groups can induce the hetero seeding process for the aimed products, and the shell layer can thus be assembled on the surface of the core.

Another strategy for the configuration of core-shell structure is epitaxial growth from vapor phase or liquid solution^16^,^17^,^18^,^19^,^20^. In such synthesis, the lattice of the core and the shell keeps highly similarity so that lattice matches and high synergic effect can be gained. However, epitaxial growth is generally conducted by relatively expensive way, e. g. chemical/physical vapor deposition (CVD/PVD), ion implantation or molecular beam epitaxy (MBE). Substrates acting as core should be prepared prior to the deposition of shell layers. Few reports have mentioned *In-situ* epitaxial growing with very facile and cost-effective method.

Here we report an *In-situ* assembling method to configure core-shell structure by facile solvothermal synthesis based on liquid phase epitaxy. Nano crystalline shell is assembled *In-situ* on the surface of the amorphous core with similar composition. The facile way by *In-situ* synthesis with contrast amorphous/crystalline structure in the core and the shell is probably providing new strategy to configure core-shell structure.

Some transition oxides, such as TiO_2_, Nb_2_O_5_, have received intense attention for their wide application in energy transferring devices e. g. solar cells, lithium ion batteries as well as photocatalyzing fields^21^,^22^,^23^,^24^,^25^,^26^,^27^,^28^,^29^,^30^,^31^,^32^,^33^,^34^. For the preparation of TiO_2_ or Nb_2_O_5_ via different solution chemical synthesis, acid is generally the most important reagent added to control the morphology, size particle, crystalline microstructure and spatial structure of the final products^10^,^14^,^15^,^21^,^22^,^23^,^24^,^25^,^29^,^30^,^31^,^32^,^33^,^34^. In this work, we propose template-free as well as acid-free facile solvothermal synthesis of Nb_2_O_5_/TiO_2_ core-shell microspheres. Liquid epitaxy induced by the changes in chemical circumstance during the process of co-precipitation is the dominant reason for triggering *In-situ* self–assembling behaviour for the fabrication of core-shell architecture.

## Results

### The process for *In-situ* fabricating core-shell architecture

**Figure 1:**
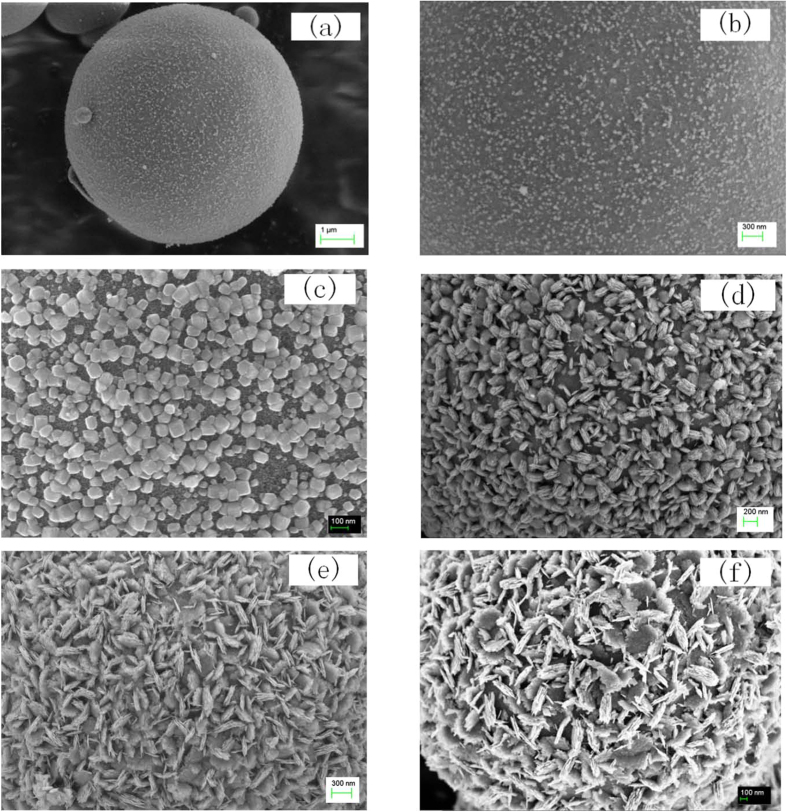
The SEM images of as-prepared core-shell samples from solvothermal synthesis at 170 °C by controlling the reaction time of (**a**,**b**) 10 h with different amplifications, (**c**)15 h, (**d**) 20 h, (**e**) 40 h and (**f** ) 60 h.

The preparation of TiO_2_ is sensitive to pH value, so adding acid or utilizing buffer solution to stabilize the chemical circumstances to tune the morphology and microstructure of the resulted TiO_2_ is a conventional way to synthesize TiO_2_-containing composites. In this report, on the contrary, we utilized the acid sensitivity of the reaction and didn’t add any additives, so that the morphology and the microstructure of the products can be controlled spontaneously by the dramatic changes in chemical circumstances during reaction. Liquid phase epitaxy can thus be induced *In-situ*. Figure 1 is the SEM images of core-shell Nb_2_O_5_/TiO_2_ composites prepared by template-free as well as acid-free facile solvothermal synthesis. The experiments were conducted at 170 °C with different reaction time. When the reaction time is controlled to 10 hours, some scattered fine particles begin to grow on the surface of the initially formed spheres, although they scattered scarcely (Fig. 1a,b). With different reaction time, the size distribution of the composite microspheres is almost kept similar except for the morphology of the surface layer, so the images given in Fig. 1c–f is the morphology of the counterpart surface of one individual core-shell microsphere, respectively. Shell particles exhibit different morphology by controlling the reaction time and preferential orientation can also be found when prolonging the reaction time (Fig. 1c–f). Cubic particles are observed on the surface of the sample produced with 15-hour reaction time. When the reaction time is prolonged from 15 hours to 20 hours, the cubic particles dispersed in the shell change to multilayer structure with a macro morphology maintaining in cube, presenting the preferential orientation growth. When the reaction was prolonged to 40 hours or 60 hours, the hierarchical laminate structure becomes much more obvious like petals and the surface of the “core” microspheres is covered overall (Fig. 1e,f).

**Figure 2:**
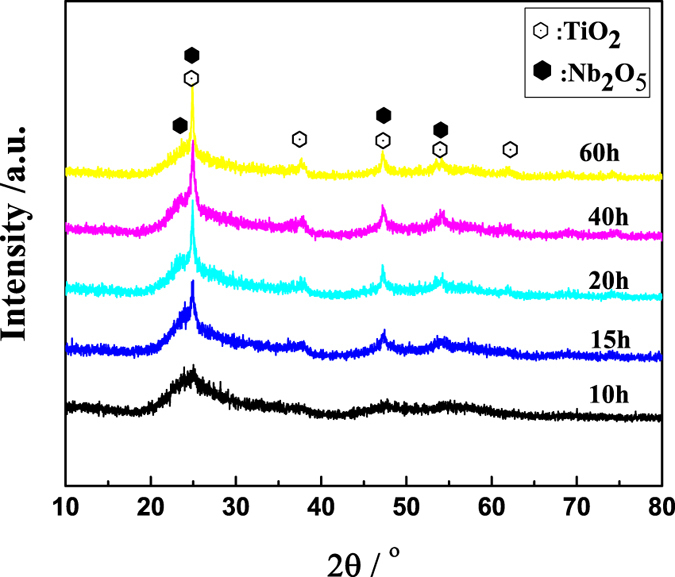
The XRD profiles for the as-prepared core-shell samples from solvothermal synthesis at 170 °C by controlling the reaction time.

**Figure 3:**
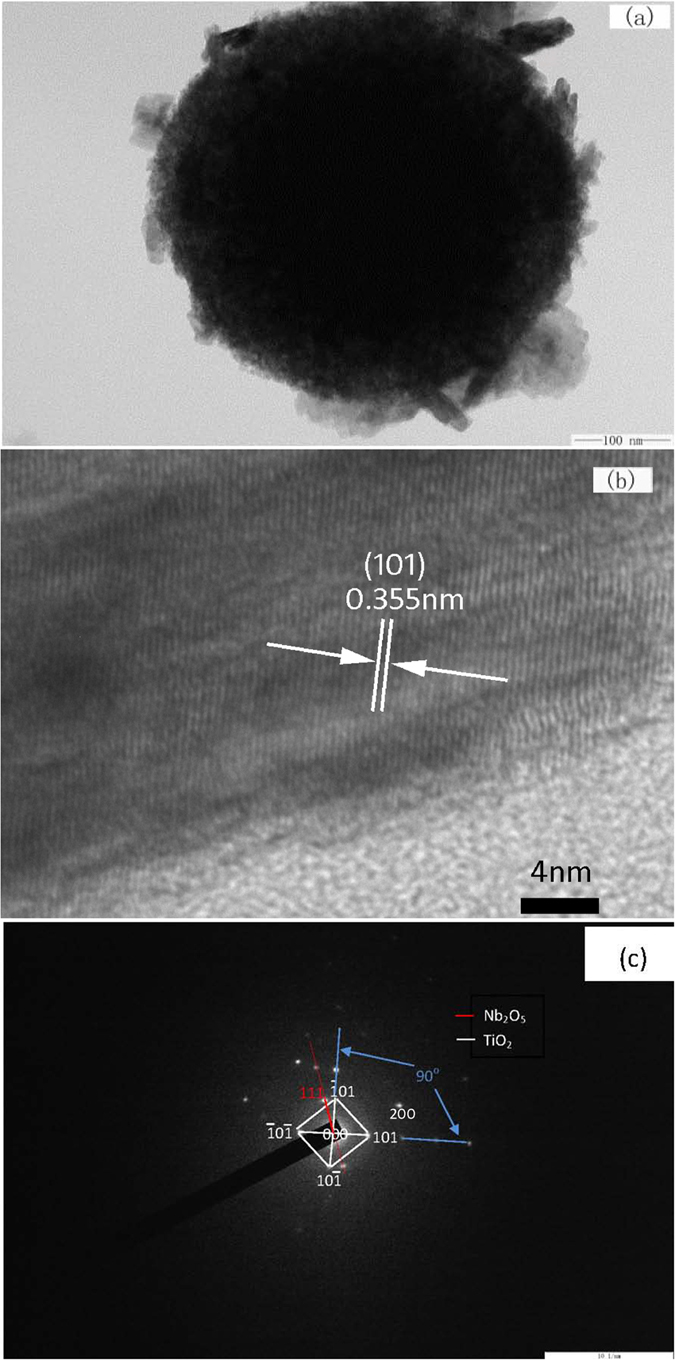
TEM images of core-shell Nb_2_O_5_/TiO_2_ composite produced at 170 °C for 20 hours (**a,b**), and the SEAD image of the shell (**c**), the scale bar of (**c**) is 10 1/nm.

**Figure 4:**
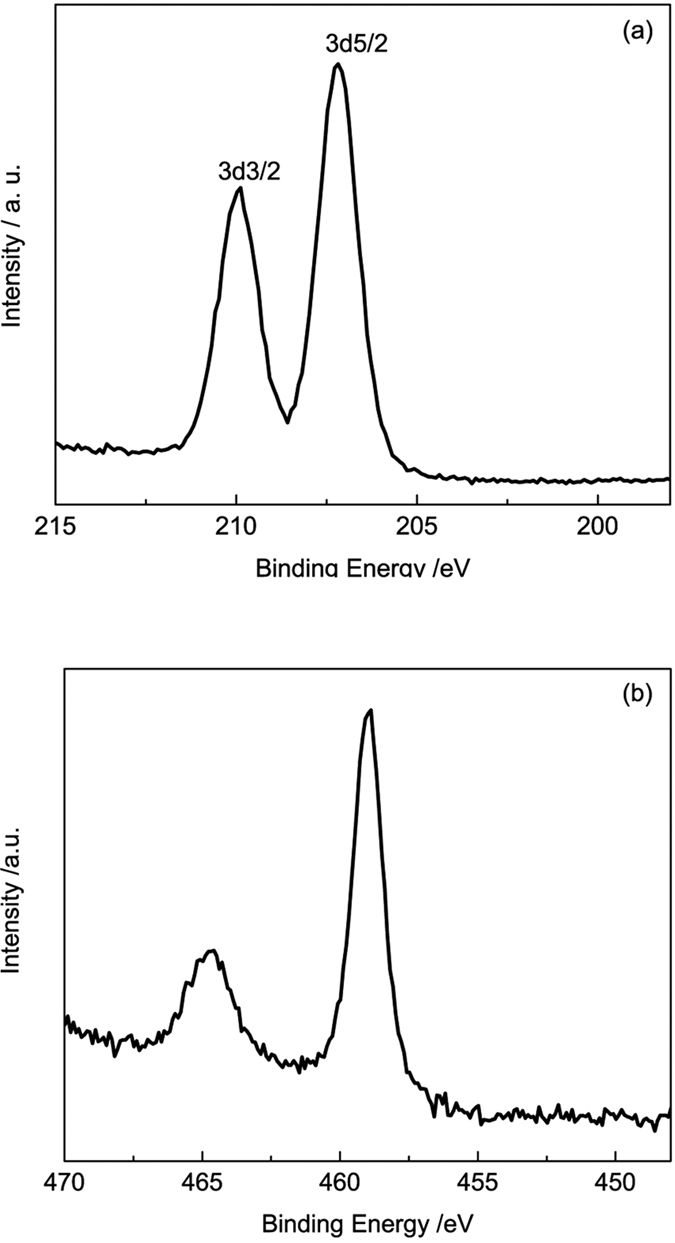
The XPS analysis for as-prepared sample with reaction time of 5 hours: (**a**) The XPS profiles for Nb 3d and (**b**) The XPS profiles for Ti 2p.

X-ray diffraction measurement was performed to detect the microstructure of the synthesized core-shell particles with different reaction time. The results are shown in Fig. 2. Broadened peaks are found in the diffraction profiles when the reaction time is relatively shorter, attributing to the amorphous structure of the products yielded with 10 hours (Fig. 2). Sharp diffraction can be observed when prolonging the reaction time, corresponding to the diffraction of anatase TiO_2_ and Nb_2_O_5_. Combining the morphology of the synthesized particles shown in Fig. 1, it is interesting to note that the intensity changes of the strong lines in the XRD profiles are simultaneously consistent with the morphology changes in shell where fine particles in regular crystalline shape are formed densely with prolonged reaction time. For example, when the reaction time was prolonged to 15 hours, the scattered fine crystalline particles on the surface of the initially formed microspheres become into cubic. Correspondingly, the corresponding XRD intensity at 2θ = 25° is observed enhanced. It is rational to deduce that the sharp peaks of Nb_2_O_5_/TiO_2_ are attributed the crystalline shell and the broadened peaks are owing to the amorphous core. This can be further verified by TEM measurement (Fig. 3). Figure 3 presents the TEM images and the selected area diffraction (SEAD) pattern of the sample prepared at 170 °C for 20 hours. Core-shell structure can be observed clearly in Fig. 3a, where the nano surface layer is coating on the micro core sphere. The HRTEM image of the selected area (Fig. 3b, the square in Fig. 3a) and the SEAD pattern of the shell layer presented here (Fig. 3c) proves that the surface layer composed of TiO_2_/Nb_2_O_5_ with high crystallization, due to the scattered dots (Fig. 3c). Combined the results of the HRTEM image and the SEAD patterns, the preferential orientation of TiO_2_ has been found along the direction of lattice (101) of TiO_2_. This is corresponding to the results of XRD analysis. The composition of the shell can also be verified by XPS analysis (Fig. 4). Figure 4a reveals that the binding energy at 204.3 eV and 207.4 eV represents Nb 3d3/2 and Nb 3d5/2 in Nb_2_O_5_ respectively. The peaks at the binding energy of 465.0 eV and 458.70 eV are the peaks of Ti2p in TiO_2_ (Fig. 4b).

**Figure 5:**
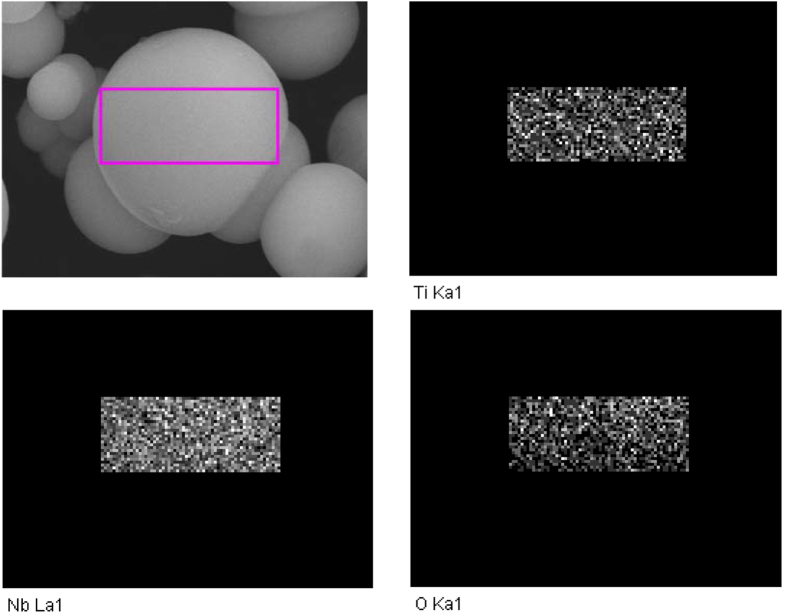
The elemental mapping images for Nb, Ti and O at the core before the formation of the shell layered.

To reveal the composition of the “core”, contrast experiment is conducted with shorter reaction time of 5 hours to obtain smooth spheres without any surface layer (seen Fig. 5). The smooth spheres practically act as the matrix for the growth of shell layer when prolonging the reaction time. The distribution of Nb/Ti in the core can be detected by elemental mapping (Fig. 5), demonstrating the homogeneous mixing state of Nb_2_O_5_ and TiO_2_ in the samples. Combined with the XRD results, it can be concluded that the shell of the core-shell composite is composed of homogeneous TiO_2_ and Nb_2_O_5_ nanocrystals, and the core is composed of amorphous TiO_2_/Nb_2_O_5_.

### Mechanism of forming core-shell structure via liquid phase epitaxy

**Figure 6:**
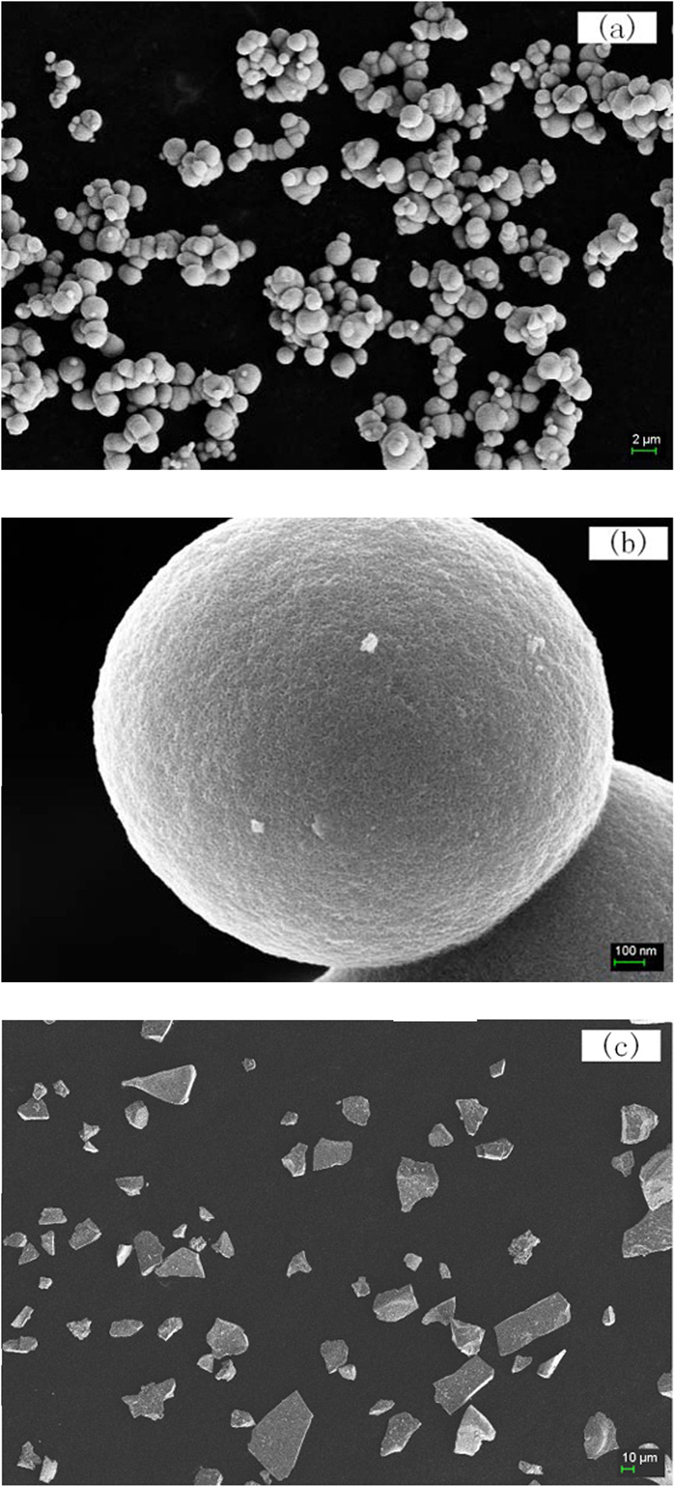
SEM images of the products from NbCl_5_ (**a,b**) and TBT (**c**) as the only reactant, respectively.

It is necessary to make an insight into the synthesis process to understand the function of each precursor. Therefore, solvothermal reaction with different precursors was conducted. NbCl_5_, TBT was used as the precursor respectively by keeping the other conditions as same as the typical process mentioned in the Methods part. Smooth microspheres could be formed when applying NbCl_5_ as the precursor (Fig. 6a,b). However, when TBT was used as the only precursor, white gel was formed during the solvothermal process. The gel was moved into oven at 120 °C, and white bulk material could be obtained finally. The SEM image of the milled products from TBT is shown in Fig. 6c. This demonstrates that the morphology of the “core” can be mainly attributed to the alcoholysis of NbCl_5_.

**Figure 7:**
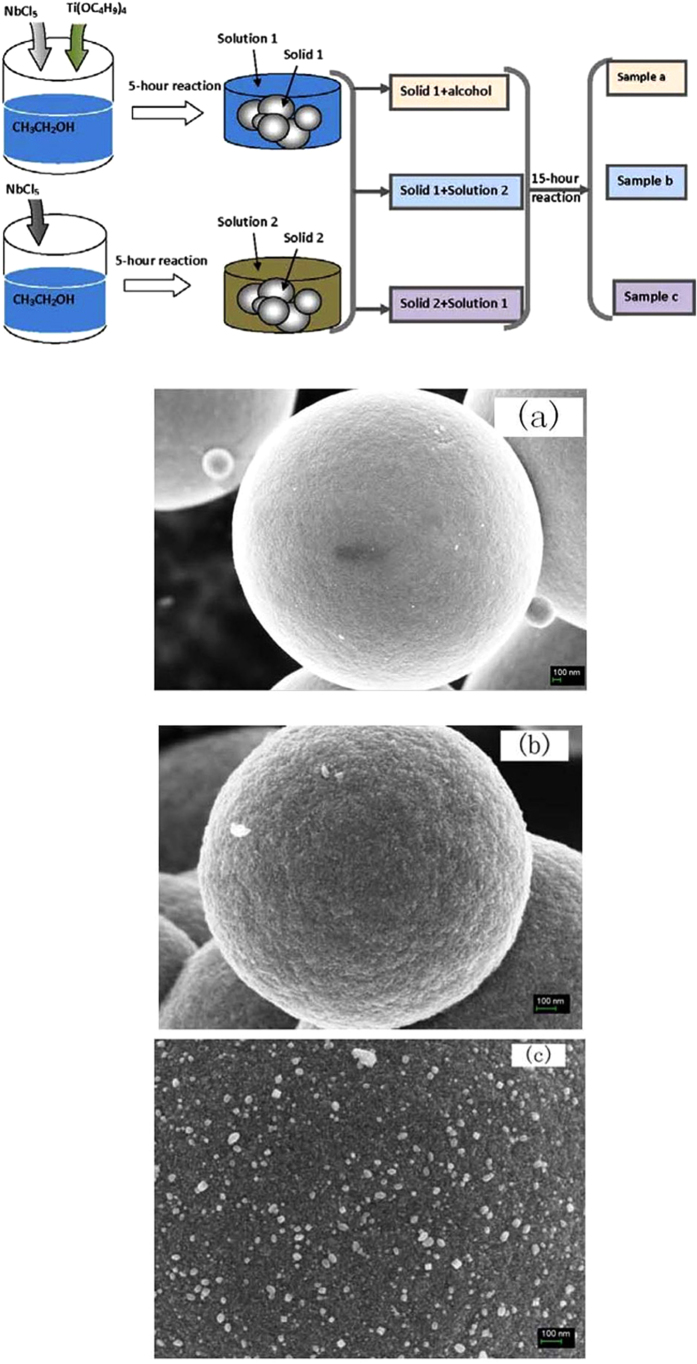
The schematic illustration of the reaction process and the SEM images of the corresponding sample (**a**) sample b and sample (**c**). The reaction is the solvothermal reaction at 170 °C. The alphabet in the SEM images is corresponding to the sample number.

It is interesting to note that the morphology change of the shell layer is accompanied with reaction time increasing, so two possibilities exist: The coating layer is crystallized from the solvent, or on the contrary, from the initially formed sphere matrix. Experiments (see Methods) were conducted following the steps as schemed in Fig. 7 to explore the possible mechanism for the formation of core-shell architecture. The morphology of sample a is correspondingly shown in Fig. 7a. Figure 7a presents that the sample a is sphere in shape with a very smooth surface, and no surface coating layer is observed, demonstrating that the “shell” layer of the core-shell composite in Fig. 1 is precipitated and crystallized from solvent instead of re-crystallized from the pre-formed bulk microspheres. Figure 7b,c is the morphology of sample b and sample c respectively. Sample b possesses a relatively clean surface (Fig. 7b), comparing to the counterpart of sample c (Fig. 7c), where fine cubic particles dispersed scarcely on the surface of the microspheres of TiO_2_/Nb_2_O_5_. Figure 7b demonstrates that the further precipitation cannot be induced for the lack of homostructured matrix for liquid phase epitaxy although the solution can be similar as used for samples shown in Fig. 1. Figure 7c demonstrates that seeding behaviour has been triggered in the bulk surface of pre-formed TiO_2_/Nb_2_O_5_. However, the lake of co-precipitation solution for TiO_2_/Nb_2_O_5_ definitely buffers the shell formation as the reaction solution was changed to NbCl_5_/alcohol residue solution.

From above metioned, it is interesting to found that the shell could not be formed well despite with same solution as the typical synthesis once the core was changed to amorphous Nb_2_O_5_. This means that the nucleation was actually taken place in the surface of the core instead in the solution, and the solution concentration was not high enough to trigger the nucleation of the shell on the surface of the core. That is, the amorphous Nb_2_O_5_/TiO_2_ core essentially functions as the matrix for shell growth and nucleation spots for shell. However, further growth was yet induced even with Nb_2_O_5_/TiO_2_ core when the solution was changed to NbCl_5_ solution. That is, the co-existence of Ti and Nb ions is another necessary factor to trigger the growth of the shell seeds. Therefore, the co-existence of Ti and Nb ions in the reaction solution is also another critical factor for liquid phase expitaxy.

**Figure 8:**
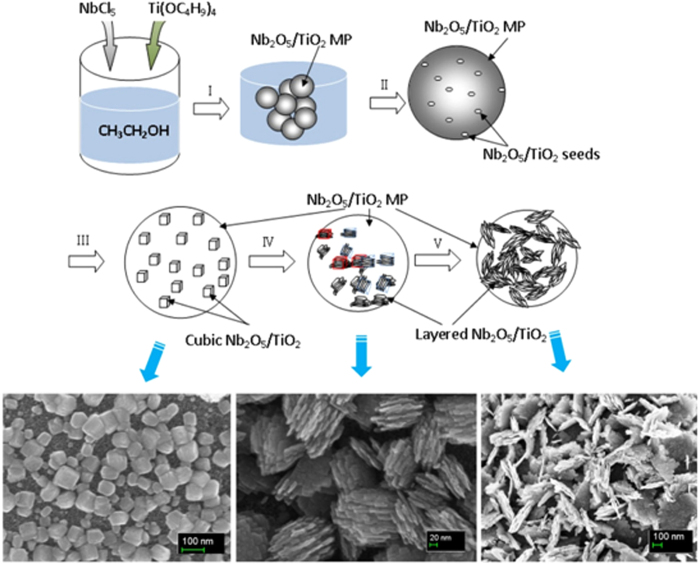
Schematic illustration of a tentative mechanism for the formation of core-shell Nb_2_O_5_/TiO_2_ composite.

Accordingly, a tentative mechanism for assembly process of core-shell Nb_2_O_5_/TiO_2_ composite microspheres with nanocrystalline Nb_2_O_5_/TiO_2_ as shell layer and spherical amorphous Nb_2_O_5_/TiO_2_ as core was proposed as illustrated in Fig. 8. The microsphere in Fig. 8 is abbreviated as “MP”.

In step I, spherical Nb_2_O_5_/TiO_2_ particles can be formed at the initial phase accompanied by the alcoholysis of NbCl_5_ and TBT when the pellucid solution transferred into autoclaved vessel is heated to high temperature. As mentioned above, spherical TiO_2_ cannot be formed when TBT is used as the only precursor at the same reaction conditions. During the time-dependent experiment (the results shown in Fig. 1), we found that the pH value decreased slightly along with reaction time being prolonged. Therefore, it is rational to deduce that the alcoholysis of NbCl_5_ definitely changes the chemical circumstances around, and the acid groups of –OCl produced by the alcoholysis of NbCl_5_ is unavoidably. The strong acid circumstances facilitate considerable co-precipitation of TBT. The oversaturation of TiO_2_ and Nb_2_O_5_ is thus taken placed and facilitates the dramatic precipitation, so the “core” is formed with homogeneously mixed TiO_2_ and Nb_2_O_5_. With the great amount of precipitation of Nb_2_O_5_/TiO_2_, the concentration of the reaction precursors decreased dramatically, which makes the further nucleation and subsequent crystal growth become difficult, so the nucleation can thus only be triggered on the surface of initially produced Nb_2_O_5_/TiO_2_ microspheres by epitaxial growth because of the relatively higher concentration and lower pH around the surface from adsorption (Step II). With the reaction time elongating accompanied with pH value dropping, the nucleation and subsequent growth of Nb_2_O_5_/TiO_2_ on the surface becomes obviously. Cubic particles can thus be observed on the surface of Nb_2_O_5_/TiO_2_ microspheres, and core-shell architecture is formed initially (Step III). According to the mechanism of Wullf construction, the surfaces with high reactivity usually diminish rapidly during the crystal growth. The preferential orientation that we observed in our process is the result of the minimization of surface energy during crystal growth. Furthermore, according to the mechanism of Orientation Attachment (OA), the growth direction can be controlled by the variation of the chemical functional group attached to the surface of crystal^34^,^35^,^36^,^37^. Preferential orientation in the special lattice direction becomes dominant with this assembling proceeding, and laminate morphology rooted from cubic crystal emerges for the preferential lattice growing faster than other lattice (Step IV). With the reaction time prolonged, the crystal growth on the preferential direction becomes more obvious and thus presents the multilayered subunits in the size of ca. 100 nm on its longitude. Hierarchical structure in the shell particles is formed (referred to Methods).

### Core-shell architecture prepared at different temperature via liquid phase epitaxy

**Figure 9:**
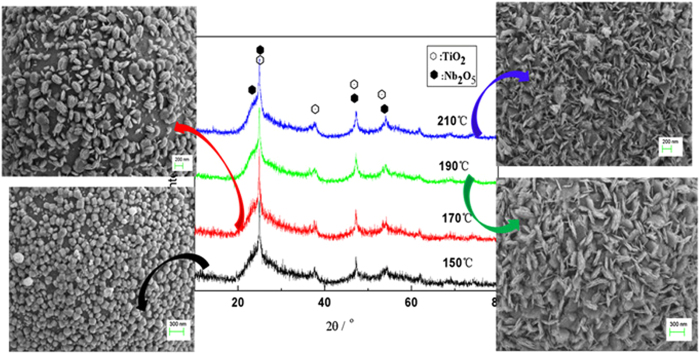
The XRD profiles and the corresponding morphology of the as-prepared samples produced at different temperature for 20 hours. The scale bar of the upper two is 200 nm and the one of the bottom two is 300 nm respectively.

As the morphology of the shell in this core-shell architecture can be controlled by the reaction time, it is deduced the similar assembling process is possibly taken place by increasing reaction temperature with shorter time. The morphology of the as-prepared deposits synthesized at different temperature by keeping the other conditions unchanged is presented in Fig. 9. It is interesting to note that the similar core/shell architecture can be formed and the morphology of the counterpart is very similar with the samples obtained from longer reaction time as shown in Fig. 1. For example, the cubic crystal on the surface of the composite formed at 150 °C for 20 hours (SEM image at the bottom right in Fig. 9) can also be observed on the sample formed at 170 °C for 15 hours (Fig. 1c). Similarly, the petal-like crystals at the counterpart from 190 °C for 20 hours (SEM image at the bottom right in Fig. 9) are consistent with the ones on the sample obtained at 170 °C for 40 hours (Fig. 1e). That is, with the temperature rising, the crystalline growth along the preferential orientation becomes obvious, showing similar behaviour in morphology changes with that by prolonging reaction time at lower temperature.

## Discussion

Nb_2_O_5_/TiO_2_ composite with core-shell architecture and hierarchical structure is obtained *In-situ* by a facile template-free and acid-free solvothermal synthesis based on the mechanism of liquid phase epitaxy. Heterostructured composite with nanocrystalline shell and amorphous core can be gained by this way. The tentative mechanism for the formation of the composite was investigated. The dramatic alcoholysis of NbCl_5_ has been demonstrated to be the fundamental factor for the formation of the spherical core, which changes the acid circumstance of the solution and induces the co-precipitaion of TiO_2_. The homogeneous co-existence of Nb_2_O_5_ and TiO_2_ in the core, which functions essentially as the nucleation spots for shell and the matrix for shell growth, facilitates the *In-situ* liquid phase epitaxy. The seeding behavior on the amorphous core and the changes in chemical circumstance during the process of co-precipitation are the dominant reason for the formation of core-shell architecture. *In-situ* liquid phase epitaxy can offer a different strategy to establish core-shell architecture for oxide materials.

## Methods

### Core-shell structure synthesis

Tetrabutyl titanate (TBT) and NbCl_5_ were used directly without further purification. The core-shell Nb_2_O_5_/TiO_2_ composite was simply achieved by the solvothermal reaction of TBT-NbCl_5_ mixed solution in ethanol. The experiments were conducted at 170 °C with different reaction time. The typical synthesis processing is as follows: 3.4 g TBT and 5.1 g NbCl_5_ were dissolved in 80 ml ethanol (A.R.) orderly to get TBT-NbCl_5_ alcoholic solution. This solution was then transferred into a Teflon-lined autoclave with an internal volume of 100 ml and subsequently heated at 170 °C for 20 hours. Suspension with white powders was gained. The produced suspension was then filtered to get white powder, which was subsequently washed by alcohol for at least 5 times. The final products were dried under vacuum at 100 °C for 8 hours. Figure 1 are the morphology of the samples with different reaction time. The SEM images in Fig. 8 are the morphology of the samples prepared with 15-, 20-, 60-hour reaction time, respectively.

### The exploration for the formation of core-shell architecture

The experiments schemed in Fig. 7 is to explore the possible mechanism for the formation of core-shell architecture. In our pervious researches, we found that smooth amorphous microspheres free of shell layer could be formed when the solothermal reaction proceeded for 5 hours. Therefore, for preparing sample a, the process of solvothermal synthesis was paused when reaction was proceeded for 5 hours by keeping the other conditions same with the above mentioned conditions. Then the reaction solution with 5-hour reaction time was put off totally, and the solid product was subsequently washed with alcohol for at least 5 times to make sure the surface of the formed microspheres was cleaned. After that, the cleaned products were removed into the reactor again and added alcohol with a regular volume of 80 ml. Then the autoclave was heated again for another 15 hours to keep the total reaction time consistent with 20 hours. The precipitates for this process is marked as sample a. The morphology of sample a is correspondingly shown in Fig. 7a. Sample b and sample c were prepared as follows: 5.1 g NbCl_5_ and NbCl_5_ (5.1 g)/TBT(3.4 g) were used as the precursors for solvothermal reaction respectively, and the reaction was conducted with the same conditions at 170 °C. After heating for 5 hours, the experiments were paused, and then the residue solution of the two reactions were exchanged with each other. Subsequently, the two reaction was continued for another 15 hours. The precipitates of the two reaction process were cleaned and dried at 100 °C. The products from NbCl_5_ and NbCl_5_/TBT were designated as sample b and sample c respectively. Figure 7b,c is the morphology of sample b and sample c respectively.

### Temperature-dependent solvothermal reaction for core-shell composite

Similar assembling process was conducted at different reaction temperature by keeping the other reaction conditions same to the above core-shell structure part. The reaction temperature was 150 °C, 170 °C, 190 °C, 210 °C, respectively. The results are shown in Fig. 9.

### Characterization of Morphology and microstructure

X-ray diffraction (RigakuD/MAX-3A), Scanning electron microscope (SEM) (SUPRA55ASAPPHIRE, Zeiss Co.) and transmission electron microscope (TEM) (JEM2100, JEOL Co.) analysis were performed to characterize the morphology and structure of as-prepared samples. X-ray Photoelectron Spectroscopy (K-Alpha 1063, Thermo Fisher Scientific Co.) was conducted to characterize the components of the surface of the samples.

## Additional Information

**How to cite this article**: Wen, Z. and Wang, G. *In-situ* Liquid Phase Epitaxy: Another Strategy to Synthesize Heterostructured Core-shell Composites. *Sci. Rep*. **6**, 25260; doi: 10.1038/srep25260 (2016).
